# The immunoregulatory mechanisms of carcinoma for its survival and development

**DOI:** 10.1186/1756-9966-30-12

**Published:** 2011-01-21

**Authors:** Caigan Du, Yuzhuo Wang

**Affiliations:** 1 Department of Urologic Sciences, University of British Columbia, Vancouver, BC V5Z 1M9, Canada; 2 Immunity and Infection Research Centre, Vancouver Coastal Health Research Institute, Vancouver, BC V6H 3Z6, Canada; 3 Vancouver Prostate Centre, Vancouver, BC V6H 3Z6, Canada; 4 Living Tumor Laboratory, BC Cancer Agency, Vancouver, BC V5Z 1L3, Canada

## Abstract

The immune system in patients detects and eliminates tumor cells, but tumors still progress persistently. The mechanisms by which tumor cells survive under the pressure of immune surveillance are not fully understood. This review is to present the evidence from clinical studies, showing a significant correlation of clinicopathological features of carcinoma with: (1) the loss of classical human leukocyte antigen class I, (2) the up-regulation of non-classical human leukocyte antigen class I, pro-apoptotic Fas ligand and receptor-binding cancer antigen expressed on SiSo cells I, and (3) the formation of immunosuppressive microenvironment by up-regulation of transforming growth factor-beta, Galectin-1, inhibitory ligand B7s, indoleamine 2,3-dioxygenase and arginase, as well as by recruitment of tumor-induced myeloid-derived suppressor cells and regulatory T cells. All of these factors may together protect carcinoma cells from the immune-cytotoxicity.

## Introduction

Carcinoma is the most commonly type of cancer transformed from epithelial cells. It has been noted for a while that the immune-mediated spontaneous regression of cancer occurs in patients [1]. Recent clinical studies have demonstrated that anti-carcinoma immunity is activated along with rise and progression of carcinoma, indicated by: (1) the tumor-infiltrating immune cells (TICs), including T, B and natural killer (NK) cells, are activated [2-4], and the number of these lymphocytes and macrophages positively correlates with cancer-specific survival rate in patients with various carcinomas [5-7]; (2) both carcinoma antigen-specific cytotoxic T lymphocytes (CTLs) [8-10] and antibodies [11-13] have been identified in cancer patients; and (3) spontaneous regression has been noted in many patients with carcinoma cancers, in which the number of infiltrating immune cells, including activated CD3^+ ^T cells, NK cells, antigen presenting cells (APCs), is significantly higher than that in non-regressing controls [14-16]. Therefore, the number of infiltrating immune cells becomes a reliable biomarker for predicting cancer relapse [17,18]. All these studies suggest that the immune surveillance against carcinoma is active in patients, but how carcinoma cells still can survive and grow in some patients is not fully understood. In this review, we attempted to summarize the evidence of anti-immune functions of carcinoma from both clinical and experimental studies.

### Avoidance of cytotoxic lymphocyte stimulation by attenuation of human leukocyte antigen class (HLA) molecules

#### Loss of HLA class I for avoidance of CD8^+ ^CTL activation

Classical HLA class I constitutively expresses on epithelial cells and many carcinoma cell lines, such as non-small cell lung cancer (NSCLC) [19]. Given a central role of HLA class I in the restriction of CD8^+ ^CTL recognition of carcinoma-specific antigens, loss of HLA class I expression undoubtedly becomes a major escape pathway for the evasion of CD8^+ ^CTL surveillance, by which any HLA class I deficient carcinoma variants can develop to more aggressive or invasive phenotypes without stimulation of primary anti-carcinoma immunity, CD8^+ ^T cell response. Indeed, as listed in Table 1, the total loss of HLA class I expression is more frequently noted with more aggressive or metastatic stages and poor differentiation phenotypes as compared to those with early stages and well to moderately differentiated lesions in patients.

**Table 1:** The association of deficient HLA class I expression in carcinoma with its progression in patients

Carcinoma type	Antibodies for immunohistochemical staining	Distribution of total HLA class I expression loss (% of negative staining*)	References
Bladder	W6/32 and GRH1	The altered of HLA class I including total losses associates with higher grade lesions and tumor recurrence	[20]
	A-072	1) 16.6% in G1, 38.5% in G2, and 57.1% in G3; 2) 5-year survival: 74% with positive versus 36% with negative staining	[21]

Gastric	A-072	0% in T1 (mucosa & submucosa) versus100% in T2-3 (muscle and fat invasion)	[22]

Esophageal	W6/32	0%: normal and benign versus 40.5% carcinoma lesions	[23]

Bronchogenic	W6/32 and HC-10	1) 13% of Diploid versus 45% of Aneuploid; 2) 17.3% in G1-2 versus 69% in G3	[24]
NSCLC	W6/32	1) 26.8% in T1-2 versus 35% in T3; 2) 20.7% in G1-2 versus 39.3% in G3; 3) 24.1% in N0 versus 34.5% in N1-2	[25]

Breast	HC-10	0% in low-grade versus 67.6% in high-grade lesions	[26]
	W6/32	24% in primary versus 64% in corresponding LN samples	[27]

Pancreatic	W6/32 and 246-E8.E7	1) 6% in primary versus 43% in metastastic tumors; 2) 0% in G1, 33% in G2 and 67% in G3	[28]

Prostate	A-072	1) 0% in Benign, 41% in primary and 66% in LN metastases; 2) 33% in low-grade versus 50% in high grade lesions	[29]

*The cutoff line for negative staining or total loss is 5 to 25% of cells stained with antibodies. W6/32 monoclonal antibody (mAb) detects monomorphic epitope of HLA class I antigen (HLA-ABC); 246-E8.E7, HC-10 and GRH1 are anti-beta2-microglobulin (β2-m) mAbs; rA-270 is rabbit polyclonal anti-β2-m antibody (DAKO).

A higher level of HLA class I expression in bladder carcinoma is significantly associated with a longer survival rate in patients [21], and tumors with a normal level of HLA class I harbor more CD8^+ ^T cells than those with altered HLA class I in renal cell carcinomas (RCC) [30] and cervical carcinoma [31,32]. In addition, a decrease in HLA class I expression has been noted as early as in normal mucosa surrounding the tumor or in situ lesion, and is significantly associated with subsequent development to a new primary tumor lesion [33,34]. These data indicate that the avoidance strategy may occur at early stages of carcinoma development, and suggest that by loss of HLA class I expression to avoid CD8^+ ^CTL seems critical for the development of carcinoma in patients.

#### Heterogeneous expression of HLA class I in inactivation of NK cell cytotoxicity

Although loss of HLA class I may benefit to carcinoma resistance to CD8^+ ^CTL as discussed above, it could increase the susceptibility to cytotoxicity of natural killer (NK) cells [35] because HLA class I is a ligand for inhibitory receptor family, killer cell immunoglobulin-like receptor (KIR) of NK cells [36], Thus, loss of HLA class I expression could favor the escape of antigen-dependent cytotoxicity of CD8^+ ^CTL, but at the same time carcinoma cells may become a target of NK cell cytotoxicity. To date, it is not completely clear how carcinoma cells can survive under the selection of both CD8^+ ^CTLs and NK cells simultaneously. It has been suggested that carcinoma cells find a balance between maintenance of HLA class I expression for inhibition of NK cell cytotoxicity and loss of its expression for the escape from CD8^+ ^CTL responses. Indeed, the complete loss of HLA class I is barely seen in carcinomas, which may be explained by its need for inhibition of NK cell activity. The heterogeneous losses of HLA class I either positively or negatively correlate with carcinoma stages or grades in patients [24,27,28], reflecting exactly the situation of carcinoma cells; if carcinoma cancer faces more severe cytotoxicity from NK cells versus CD8^+ ^CTL, certain levels of HLA class I render carcinomas resistance to NK cells; if tumor is under the pressure of CD8^+ ^CTL more than NK cells, then partial loss of HLA class I becomes a key for survival, as indicated by Table 1.

In addition to heterogeneous expression of HLA class I, one has to knowledge that other strategies are seen to avoid NK cell cytotoxicity. A clinical study with oral squamous cell carcinomas shows that HLA class I expression is either weak or absent for not stimulation of CD8^+ ^CTL, but there is still no a clear correlation of HLA class I expression loss with a relative proportion of NK cells, indicating that the local factors seem to down-regulate the final outcome of the cytotoxic immune response of NK cells [33]. Indeed, reduced expression of natural cytotoxicity receptor, NKG2D ligand UL16 binding protein 1 and Inter-Cellular Adhesion Molecule 1 has been seen on tumor cells [37,38], which may specifically prevent NK cell activation.

#### Non-classical HLA-G in inhibition of both CD8^+ ^CTLs and NK cells

HLA-G is a non-classical class I antigen, originally detected in trophoblastic cells [39], where it is proposed to suppress maternal immune response against the semi-allogeneic fetus. It binds to the inhibitory receptors Ig-like transcript (ILT) 2, ILT4 or KIR2DL4, resulting in suppression of cytotoxicity of both CD8^+ ^CTL and NK cells [40,41]. The protective role of HLA-G in carcinoma survival under immune surveillance is demonstrated in many studies with patients; in contrast to its null expression in normal epithelial cells and benign adenomas, a high percentage (30-90%) of carcinoma cells expresses HLA-G in a variety of cancerous lesions, and its levels have been found to be significantly associated with clinicopathological features and shorter survival time of patients [42-45]. All these data indicate that carcinoma-expressing HLA-G could be one of important mechanisms for inhibition of both CD8^+^CTL and NK cell mediated anti-carcinoma immunity.

### Induction of TIC apoptosis by expression of pro-apoptotic ligands

#### Fas ligand (FasL)

FasL binding to death receptor Fas triggers apoptosis of Fas-expressing cells including TICs. Two patterns of FasL expression on carcinoma cells have been shown by immunohistochemical staining: (1) up-regulation of FasL expression on carcinoma is positively associated with clinicopathological features in patients, shown by that FasL expression is an early event in epithelial cell transformation (adenoma), followed by an increase in the percentage of FasL-expressing carcinoma cells in high-stage or -grade lesions, and the poorer survival of patients with high levels of FasL expression (Table 2); and (2) high levels of FasL expression have been seen as an independent factor for clinicopathological features, indicated by the positive staining of persistent FasL expression regardless of tumor stage, histologic grade, invasion and metastasis in many studies [47,58-61]. All of these observations suggest that FasL expression is critical for carcinoma survival by induction of TIC apoptosis. Indeed, the pro-apoptotic function of FasL on carcinoma cells has been demonstrated in both in vitro and in vivo; in co-cultures with a variety of carcinoma cell lines, FasL expressed on carcinoma cells induce apoptosis of lymphocytes in Fas-dependent manner [49,51,62-66], and in carcinoma biopsies from patients, the present of FasL on carcinoma cells is in parallel with apoptosis of TICs [53,60,67-69] or reduced number of TICs [70,71]. In the experimental studies with animal models, down-regulation of FasL expression in carcinoma significantly reduces tumor development in syngeneic immunocompetent mice [72], while persistent expression of Fas enhances tumor growth along with an increase in lymphocyte apoptosis [73,74], and is acquired for survival from active specific immunotherapy [75].

**Table 2:** FasL expression in carcinoma cancers

Carcinoma type	Distribution of high FasL expression	References
Colorectal	19% in adenomas, 40% of stage I-II, 67% of stage III and 70% of stage IV of carcinoma	[46]
	40.9% in adenoma versus 80.8% in carcinoma	[47]
	Higher incidence of metastases and poorer patients' survival associate with FasL positive carcinomas	[48]
	0 positive in normal epithelial cells, 2/7 positive in primary tumors, 4/4 positive in hepatic metastatic tumors	[49]

Adrenocortical	37.7% in adenomas versus 100% in the carcinoma	[50]

Bladder transitional cell	1) 0% in normal urothelium, 0% in G1, 14% in G2, and 75% in G3. 2) 13% in superficial Ta-T1 versus 81% in invasive T2-T4	[51]
	0% in normal urothelium, 19% in T1, 21% in T2 and 49% in T3	[52]

Pancreatic ductal	1) 82% in primary versus 100% in hepatic metastases 2) Shorter survival for patients associates with FasL positive tumors	[53]

Nasopharyngeal	1) 0% in stage I, 57% in stage II, 58% in stage III and 82% in stage IV; 2) A lower rate of disease-free and overall survival for patients associates with positive FasL expression.	[54]

Gastric	36.2% in adenomas, 68.8% in early carcinoma, and 70.4% in advanced carcinoma	[55]

Cervical	1) 5/14 in inner 2/3 stromal invasion versus 10/10 outer 2/3 stromal invasion; 2) 7/15 without LN metastasis versus 8/9 with LN metastasis; 3) Reduced survival times in patients with FasL-expressing tumors	[56]

Esophageal	1) Higher incidence of LN metastasis associates with the tumors containing >25% FasL expression; 2) All cancer metastases in LN express FasL in >50% of the cells	[57]

LN: lymph nodes

#### Receptor-binding cancer antigen expressed on SiSo cells (RCAS) 1

RCAS1 is a recently characterized human tumor-associated antigen expressed in a wide variety of cancer tissues, and induces cell cycle arrest and/or apoptosis in RCAS1 receptor-expressing immune cells. Like FasL on carcinoma cells, RCAS1 is expressed in a high percentage of carcinoma cells (30-100%) and is significantly correlated with clinicopathological features including a shorter survival time for patients, and with apoptosis or reduction of TICs [76-81]. In co-cultures of interleukin (IL)-2 activated peripheral blood lymphocytes with human oral squamous cell carcinomas cell line (KB cells), lymphocyte apoptosis is associated with the presence of soluble RCAS1 in the medium [77]. In addition, similar to FasL and RCAS1, CD70 overexpressed on RCC promotes lymphocyte apoptosis by binding to its receptor CD27, indicating a proapoptotic role of CD70 in the elimination of TICs as well [82]. All these observations suggest that the direct induction of TIC apoptosis by persistent expression of FasL, RCAS1 or perhaps other apoptosis-inducing ligands (e.g. CD70) on carcinoma cells plays a role in the ability of carcinoma cells to escape from the anti-carcinoma immunity.

### Suppression of TIC activity by molecular and cellular factors

#### Immunoregulatory cytokine/cytokine-like: Transforming growth factor (TGF)-β1 and Galectin-1 (Gal-1)

TGF-β1 is a multifunctional cytokine involved in immunosuppression. Numerous clinical studies have demonstrated that a higher level of TGF-β1 expression is significantly associated with an invasive phenotype of tumors or metastases in patients [83-86]. In vitro a significant amount of TGF-β1 is produced in the poorly differentiated prostate carcinoma cell lines but not in well-differentiated cells [87]. These data imply that TGF-β1 may increase metastasis by a paracrine matter, such as suppression of local immune response or increased angiogenesis. Indeed, in the biopsies of cervical carcinoma tumors, an inverse relationship between TGF-β1 expression in tumor cells and the extent of TICs is demonstrated [88]. This clinical observation is further confirmed by several experimental studies. In a mouse skin explant model, TGF-β1 is produced by progressor types but not regressor squamous cell carcinoma lines, and this tumor-derived cytokine inhibits migration of professional APCs, Langerhans cells (LCs), and keeps them in an immature form [89], or transgenic expression of TGF-β1 enhances growth of regressor squamous carcinoma cells in vitro and in vivo just like progressor phenotype, and reduces the number of infiltrating LCs, CD4^+ ^and CD8^+ ^T cells [90]. A further study with invasive colon carcinoma U9A cell line shows that decreasing TGF-β1 expression by antisense reduces the invasive activity and metastasis of tumor cells to the liver [91]. All these studies suggest that carcinoma-derived TGF-β plays an important role in the tumor metastasis, which may be caused by its immune suppressive function.

Gal-1 is a member of β-galacosidess binding protein family (galectins), and is a recently identified immunoregulatory cytokine-like molecule in cancer [92]. It has been documented that Gal-1 exhibits immunoregulatory effects by which it controls immune cell trafficking, regulates activation of dendritic cells (DCs) and induces T-cell apoptosis [93]. Up-regulation of Gal-1 expression has been seen in a variety of carcinoma biopsies, particularly in tumor-associated stroma, and is associated with tumor invasiveness or worse prognoses [94-97] and with reduced infiltrating T cells [98], suggesting that Gal-1, produced by carcinoma and/or stromal cells surrounding the tumor, may take a part in the carcinoma immune-escape by regulation of T cell homeostasis. This hypothesis is supported by a recent study showing that tumor cell-expressing Gal-1 induces T cell apoptosis in a co-culture system [99].

#### Immune inhibitory ligands: B7 family members (B7-H1, -H3 and -H4)

B7-H1 (PD-L1) is a ligand for the receptor PD-1 on T cell, and is known to negatively regulate T-cell activation [100]. Similar to B7-H1, B7-H3 or -H4 ligation of T cells has a profound inhibitory effect on Th1 differentiation [101], as well as the proliferation, differentiation and cytotoxicity of T cells [102]. Over-expression of these B7 family members (B7-H1, -H3 or -H4) has been documented in various types of carcinoma as compared to healthy controls: (1) H7-H1 in pancreatic tumors [103,104], RCC [105,106], human hepatocellular carcinoma (HCC) [107,108], urothelial cell carcinoma (UCC) [109] and NSCLC [110]; (2) B7-H3 in UCC [111]; and (4) H7-H4 in NSCLC [112], breast cancer [113,114] and ovarian cancer [115]. Tumor B7-H1 expression is significantly associated with less TICs including PD-1 positive immune cells, poor tumor differentiation, advanced tumor stage and poorer survival of patients [103,104,106-110,115]. Similar correlation of B7-H4 with clinicopathological features has been reported as well [111-114].

In parallel with up-regulation of B7-H1, the number of PD-1^+ ^CD8^+ ^cells increases in tumor tissues, such as HCC [108,116] and prostate cancer [117], and these tumor-infiltrating CD8^+ ^cells have been shown to be impaired in the granule and cytokine productions [108,117-119]. In addition, blocking the interaction of B7-H1 with PD-1 using neutralizing antibody restores the effector function of tumor-infiltrating T cells [108,119] and in a mouse model of pancreatic cancer, the antibody therapy, combined with gemocitabine, induces a complete regression of tumor growth [104]. All these studies indicate that up-regulation of B7 inhibitory molecules acts as an immunosuppressive strategy for carcinoma to escape from anti-carcinoma immunity during cell-cell contact with T cells.

#### Depletion of amino acids enzymes: indoleamine 2,3-dioxygenase (IDO) and arginase (ARG)

The mechanisms by which IDO induces immunosuppression have been recently reviewed [120]. IDO is a tryptophan-catabolising enzyme. Up-regulation of its synthesis has been documented in IFN-γ-stimulated cultures of KB oral carcinoma and WiDr colon adenocarcinoma [121], pancreatic carcinomal cells [122], hepatocellular carcinoma cell lines [123], and colorectal carcinoma cell lines [124]. Over-expression of IDO protein is reported in the cancerous lesions, and significantly correlates with carcinoma metastasis and poor prognosis in patients with a variety of carcinoma cancers [122-126]. The up-regulation of IDO is associated with a significant reduction of CD3^+ ^TICs [124], or with an increased number of regulatory T (Treg) cells in the metastatic carcinoma in lymph nodes (LNs) [122]. Ectopic expression of IDO enhances tumor growth of the human endometrial carcinoma cell line AMEC and suppresses cytotoxicity of NK cells in a mouse xenograft model [127]. All these observations suggest that IDO-high expression in carcinoma cells in primary tumors may defeat the invasion of effector T cells and NK cells via local tryptophan depletion as well as production of proapoptotic tryptophan catabolites. Also, IDO in metastatic carcinoma cells may enhance the differentiation of Treg cells as a potent immunosuppressive strategy.

ARG is an arginine-metabolic enzyme converting L-arginine into L-ornithine and urea [128]. It has been suggested that arginine is one of essential amino acids for T cell activation and proliferation [129], and the depletion of extracellular arginine by ARG results in the modulation of CD3ζ chain expression and proliferative suppression in T cells [130]. A significantly high level of ARG activity has been demonstrated in the carcinomas of the prostate [131], the gallbladder [132] and the lung [133,134], but the evidence for the contribution of ARG activity to tumor immune escape is still weak; ARGII and NOSII together has been shown to participate in local peroxynitrite dependent immune suppression of prostate cancer [135], but not seen in lung cancer [136]. However, this enzyme may play a critical role in the immunosuppressive activity of tumor-induced myeloid-derived suppressor cells (MDSCs) as discussed below.

#### Immunosuppressive cells: CD4^+^CD25^+^Foxp3^+ ^regulatory T (Treg) cells and Tumor-induced myeloid-derived suppressor cells (MDSCs)

Treg cells can inactivate both effector/helper T and B cells. After activation, Treg cells not only produce abundant anti-inflammatory cytokine IL-10 and TGF-β, but also express cell surface CTLA-4, which binds to B7 molecules on APCs, resulting in suppression of effector T cells and their dependent B cells. Numerous studies with cancer patients have demonstrated that the prevalence of Treg cells is significantly high in cancerous lesions as compared to those in healthy controls [136-141], and the percentage of Treg cells among TICs positively correlates with a significantly lower survival rate [138,139,142]. In mice challenged with pancreas adenocarcinoma cells (Pan02), depletion of Treg cells promotes a tumor-specific immune response, and significantly associates with smaller size of tumor and longer survival [143]. All these studies suggest that an increase in Treg cells in TICs may play a central role in self-tolerance to carcinoma cells, which may "hijack" these Treg cells as an effective strategy for immunoescape by suppression of anti-carcinoma immunity.

However, the mechanism of elevation of Treg cells in TICs is not fully clarified, but may be due to their local proliferation/differentiation or recruitment from circulation to cancerous lesion or to both. Indeed, the presence of Treg cells in carcinoma lesions is in conjunction with immature DCs, Th2 cytokine dominant microenvironment, prostaglandin E2 (PGE2) and IDO activity [122,144,145] or is required the function of CCL22 [146] and/or CCL5 [147]. Chemokine CCL22 and CCL5 mediate trafficking of Treg cells to the tumors, whereas immature DCs, Th2 cytokines and PGE2 favor Treg cell proliferation and/or differentiation.

MDSCs represent a heterogeneous population of immunosuppressive cells expressing a variety of surface markers, such as CD11c^+^, CD11b^+^, CD33^+^, CD34^+ ^and CD15^+^. In patients with all different types of carcinomas, an increasing number of MDSCs have been found in peripheral blood [148-150] and/or intratumor lesions [151-153]. The frequency of these cells also positively correlates with the incidence of recurrence or metastatic disease in patients [153,154]. Experimental studies show that MDSCs can function as potent suppressors of cytotoxicity of both effector CD8^+ ^T-cells [155] and NK cells [156]. The immunosuppressive activities of MDSCs may depend on the activity of ARG and/or reactive oxygen species they produce [150,157,158] or the induction of Foxp3^+ ^Treg cells [159]. All these studies suggest that MDSCs may be one of important factors responsible not only for systemic immune dysfunction in cancer patients but also for local carcinoma immune escape.

## Conclusions

**Figure 1:**
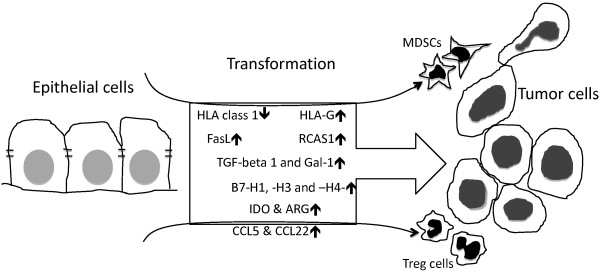
**Diagram for the expression of immunoregulatory molecules during the transformation of epithelial cells to carcinoma tumor cells under the pressure from immune surveillance**. Loss of classical and/or up-regulation of non-classical HLA class I expressions may be able to avoid the stimulation of cytotoxic CD8^+ ^T cells and NK cells; Up-regulation of pro-apoptotic ligands, such as Fas L and RCAS1 may directly induce anti-carcinoma immune cell death. Secretion of TGF-beta1 and Gal-1, expression of immune inhibitor ligands (B7-H1 -H3 and -H4), up-regulation of IDO and/or ARG activity and/or expansion of cellular immunosuppression by MDSCs and Foxp3 Treg cells could generate an immunosuppressive microenvironment, protecting carcinoma cells from immune surveillance.

The evidence from the limited literature we reviewed clearly indicates that carcinoma development in patients closely correlates to its ability to inactivate effector cytotoxic lymphocytes (i.e. CD8^+ ^CTL and NK cells), to induce TIC apoptosis and/or to suppress the anti-carcinoma immune response, as indicated by: (1) down-regulation of antigen-presenting protein HLA class I; (2) up-regulation of immunosuppressive proteins, such as cell surface FasL, HLA-G, immune inhibitory ligand B7 family members, secreted cytokine TGF-β and Gal-1, enzyme IDO and perhaps ARG, and (3) induction/expansion of immunosuppressive cells: MDSCs and/or Foxp3^+ ^Treg cells (Figure 1). Thus, it must be acknowledged that carcinoma develops multiple adaptation mechanisms against immune surveillance, but different types of carcinoma cancer may use different anti-immune strategies depending on the spectrum of host anti-carcinoma immunity in patients. Further understanding of these mechanisms by which carcinomas cells resist to anti-carcinoma immunity will lead to develop more effective immunotherapyi

## Abbreviations

APC: Antigen presenting cell; ARG: Arginase; CTL: Cytotoxic T lymphocyte; DC: Dendritic cell; Gal: Galectin; HCC: human hepatocellular carcinoma; HLA: Human leukocyte antigen; HNSCC: Head and neck squamous cell carcinoma; IDO: Indoleamine 2,3-dioxygenase; IL: Interleukin; ILT: Ig-like transcript; KIR: Killer cell immunoglobulin-like receptor; LC: Langerhans cell; MDSC: Tumor-induced myeloid-derived suppressor cell; NK: Natural killer; NSCLC: Non-small cell lung cancer; PGE2: Prostaglandin E2; RCAS1: Receptor-binding cancer antigen expressed on SiSo cells; RCC: Renal cell carcinomas; TGF: Transforming growth factor; TIC: Tumor-infiltrating immune cell; Treg: Regulatory T cel; UCC: Urothelial cell carcinoma.

## Competing interests

The authors declare that they have no competing interests.

## Authors' contributions

YW initiated the concept. CD drafted the manuscript. Both authors participated in writing, read and approved the final manuscript.

## References

[B1] Cole WH Relationship of causative factors in spontaneous regression of cancer to immunologic factors possibly effective in cancer J Surg Oncol 1976 8 391 411 10.1002/jso.2930080506 792571

[B2] Whiteside TL The role of immune cells in the tumor microenvironment Cancer Treat Res 2006 130 103 124 full_text 16610705 10.1007/0-387-26283-0_5

[B3] Maccalli C Scaramuzza S Parmiani G TNK cells (NKG2D^+ ^CD8^+ ^or CD4^+ ^T lymphocytes) in the control of human tumors Cancer Immunol Immunother 2009 58 801 808 10.1007/s00262-008-0635-x 19089424 PMC11030286

[B4] Nelson BH CD20^+ ^B cells: the other tumor-infiltrating lymphocytes J Immunol 2010 185 4977 4982 10.4049/jimmunol.1001323 20962266

[B5] Cho Y Miyamoto M Kato K Fukunaga A Shichinohe T Kawarada Y Hida Y Oshikiri T Kurokawa T Suzuoki M Nakakubo Y Hiraoka K Murakami S Shinohara T Itoh T Okushiba S Kondo S Katoh H CD4^+ ^and CD8^+ ^T cells cooperate to improve prognosis of patients with esophageal squamous cell carcinoma Cancer Res 2003 63 1555 1559 12670904

[B6] Eerola AK Soini Y Paakko P Tumour infiltrating lymphocytes in relation to tumour angiogenesis, apoptosis and prognosis in patients with large cell lung carcinoma Lung Cancer 1999 26 73 83 10.1016/S0169-5002(99)00072-0 10568678

[B7] Oberg A Samii S Stenling R Lindmark G Different occurrence of CD8^+^, CD45R0^+^, and CD68^+ ^immune cells in regional lymph node metastases from colorectal cancer as potential prognostic predictors Int J Colorectal Dis 2002 17 25 29 10.1007/s003840100337 12018450

[B8] Chikamatsu K Eura M Nakano K Masuyama K Ishikawa T Functional and T cell receptor gene usage analysis of cytotoxic T lymphocytes in fresh tumor-infiltrating lymphocytes from human head and neck cancer Jpn J Cancer Res 1995 86 477 483 7790320 10.1111/j.1349-7006.1995.tb03081.x PMC5920848

[B9] Housseau F Zeliszewski D Roy M Paradis V Richon S Ricour A Bougaran J Prapotnich D Vallancien G Benoit G Desportes L Bedossa P Hercend T Bidart JM Bellet D MHC-dependent cytolysis of autologous tumor cells by lymphocytes infiltrating urothelial carcinomas Int J Cancer 1997 71 585 594 10.1002/(SICI)1097-0215(19970516)71:4<585::AID-IJC13>3.0.CO;2-B 9178812

[B10] Verdegaal EM Hoogstraten C Sandel MH Kuppen PJ Brink AA Claas FH Gorsira MC Graadt van Roggen JF Osanto S Functional CD8+ T cells infiltrate into nonsmall cell lung carcinoma Cancer Immunol Immunother 2007 56 587 600 10.1007/s00262-006-0214-y 16924494 PMC11030057

[B11] Di Modugno F Bronzi G Scanlan MJ Del Bello D Cascioli S Venturo I Botti C Nicotra MR Mottolese M Natali PG Santoni A Jager E Nistico P Human Mena protein, a serex-defined antigen overexpressed in breast cancer eliciting both humoral and CD8^+ ^T-cell immune response Int J Cancer 2004 109 909 918 10.1002/ijc.20094 15027125

[B12] Mosolits S Steinitz M Harmenberg U Ruden U Eriksson E Mellstedt H Fagerberg J Immunogenic regions of the GA733-2 tumour-associated antigen recognised by autoantibodies of patients with colorectal carcinoma Cancer Immunol Immunother 2002 51 209 218 10.1007/s00262-002-0272-8 12012108 PMC11032830

[B13] Zeng G Aldridge ME Wang Y Pantuck AJ Wang AY Liu YX Han Y Yuan YH Robbins PF Dubinett SM deKernion JB Belldegrun AS Dominant B cell epitope from NY-ESO-1 recognized by sera from a wide spectrum of cancer patients: implications as a potential biomarker Int J Cancer 2005 114 268 273 10.1002/ijc.20716 15540228

[B14] Kerr KM Johnson SK King G Kennedy MM Weir J Jeffrey R Partial regression in primary carcinoma of the lung: does it occur? Histopathology 1998 33 55 63 9726050

[B15] Patel A Halliday GM Barnetson RS CD4^+ ^T lymphocyte infiltration correlates with regression of a UV-induced squamous cell carcinoma J Dermatol Sci 1995 9 12 19 10.1016/0923-1811(94)00344-E 7727352

[B16] Patel A Halliday GM Cooke BE Barnetson RS Evidence that regression in keratoacanthoma is immunologically mediated: a comparison with squamous cell carcinoma Br J Dermatol 1994 131 789 798 10.1111/j.1365-2133.1994.tb08580.x 7531999

[B17] Nedergaard BS Ladekarl M Thomsen HF Nyengaard JR Nielsen K Low density of CD3^+^, CD4^+ ^and CD8^+ ^cells is associated with increased risk of relapse in squamous cell cervical cancer Br J Cancer 2007 97 1135 1138 10.1038/sj.bjc.6604001 17940503 PMC2360435

[B18] Øvestad IT Gudlaugsson E Skaland I Malpica A Kruse AJ Janssen EA Baak JP Local immune response in the microenvironment of CIN2-3 with and without spontaneous regression Mod Pathol 2010 23 1231 1240 20512116 10.1038/modpathol.2010.109

[B19] Wroblewski JM Bixby DL Borowski C Yannelli JR Characterization of human non-small cell lung cancer (NSCLC) cell lines for expression of MHC, co-stimulatory molecules and tumor-associated antigens Lung Cancer 2001 33 181 194 10.1016/S0169-5002(01)00210-0 11551413

[B20] Cabrera T Pedrajas G Cozar JM Garrido A Vicente J Tallada M Garrido F HLA class I expression in bladder carcinomas Tissue Antigens 2003 62 324 327 10.1034/j.1399-0039.2003.00104.x 12974799

[B21] Levin I Klein T Goldstein J Kuperman O Kanetti J Klein B Expression of class I histocompatibility antigens in transitional cell carcinoma of the urinary bladder in relation to survival Cancer 1991 68 2591 2594 10.1002/1097-0142(19911215)68:12<2591::AID-CNCR2820681212>3.0.CO;2-L 1933807

[B22] Klein B Klein T Nyska A Shapira J Figer A Schwartz A Rakovsky E Livni E Lurie H Expression of HLA class I and class II in gastric carcinoma in relation to pathologic stage Tumour Biol 1991 12 68 74 10.1159/000217690 2028181

[B23] Rockett JC Darnton SJ Crocker J Matthews HR Morris AG Expression of HLA-ABC, HLA-DR and intercellular adhesion molecule-1 in oesophageal carcinoma J Clin Pathol 1995 48 539 544 10.1136/jcp.48.6.539 7665697 PMC502684

[B24] Redondo M Concha A Oldiviela R Cueto A Gonzalez A Garrido F Ruiz-Cabello F Expression of HLA class I and II antigens in bronchogenic carcinomas: its relationship to cellular DNA content and clinical-pathological parameters Cancer Res 1991 51 4948 4954 1654207

[B25] Passlick B Pantel K Kubuschok B Angstwurm M Neher A Thetter O Schweiberer L Izbicki JR Expression of MHC molecules and ICAM-1 on non-small cell lung carcinomas: association with early lymphatic spread of tumour cells Eur J Cancer 1996 32A 141 145 10.1016/0959-8049(95)00551-X 8695222

[B26] Vitale M Rezzani R Rodella L Zauli G Grigolato P Cadei M Hicklin DJ Ferrone S HLA class I antigen and transporter associated with antigen processing (TAP1 and TAP2) down-regulation in high-grade primary breast carcinoma lesions Cancer Res 1998 58 737 742 9485029

[B27] Saio M Teicher M Campbell G Feiner H Delgado Y Frey AB Immunocytochemical demonstration of down regulation of HLA class-I molecule expression in human metastatic breast carcinoma Clin Exp Metastasis 2004 21 243 249 10.1023/B:CLIN.0000037707.07428.ff 15387374

[B28] Ryschich E Notzel T Hinz U Autschbach F Ferguson J Simon I Weitz J Frohlich B Klar E Buchler MW Schmidt J Control of T-cell-mediated immune response by HLA class I in human pancreatic carcinoma Clin Cancer Res 2005 11 2 Pt 1 498 504 15701833

[B29] Sharpe JC Abel PD Gilbertson JA Brawn P Foster CS Modulated expression of human leucocyte antigen class I and class II determinants in hyperplastic and malignant human prostatic epithelium Br J Urol 1994 74 609 616 10.1111/j.1464-410X.1994.tb09193.x 7530126

[B30] Brasanac D Markovic-Lipkovski J Hadzi-Djokic J Muller GA Muller CA Immunohistochemical analysis of HLA class II antigens and tumor infiltrating mononuclear cells in renal cell carcinoma: correlation with clinical and histopathological data Neoplasma 1999 46 173 178 10613593

[B31] Hilders CG Houbiers JG van Ravenswaay Claasen HH Veldhuizen RW Fleuren GJ Association between HLA-expression and infiltration of immune cells in cervical carcinoma Lab Invest 1993 69 651 659 8264228

[B32] Hilders CG Munoz IM Nooyen Y Fleuren GJ Altered HLA expression by metastatic cervical carcinoma cells as a factor in impaired immune surveillance Gynecol Oncol 1995 57 366 375 10.1006/gyno.1995.1156 7774840

[B33] Cruz I Meijer CJ Walboomers JM Snijders PJ Van der Waal I Lack of MHC class I surface expression on neoplastic cells and poor activation of the secretory pathway of cytotoxic cells in oral squamous cell carcinomas Br J Cancer 1999 81 881 889 10.1038/sj.bjc.6690780 10555762 PMC2374311

[B34] Grandis JR Falkner DM Melhem MF Gooding WE Drenning SD Morel PA Human leukocyte antigen class I allelic and haplotype loss in squamous cell carcinoma of the head and neck: clinical and immunogenetic consequences Clin Cancer Res 2000 6 2794 2802 10914726

[B35] Gati A Da Rocha S Guerra N Escudier B Moretta A Chouaib S Angevin E Caignard A Analysis of the natural killer mediated immune response in metastatic renal cell carcinoma patients Int J Cancer 2004 109 393 401 10.1002/ijc.11730 14961578

[B36] Lanier LL Natural killer cells: from no receptors to too many Immunity 1997 6 371 378 10.1016/S1074-7613(00)80280-0 9133416

[B37] Doubrovina ES Doubrovin MM Vider E Sisson RB O'Reilly RJ Dupont B Vyas YM Evasion from NK cell immunity by MHC class I chain-related molecules expressing colon adenocarcinoma J Immunol 2003 171 6891 6899 14662896 10.4049/jimmunol.171.12.6891

[B38] Le Maux Chansac B Moretta A Vergnon I Opolon P Lecluse Y Grunenwald D Kubin M Soria JC Chouaib S Mami-Chouaib F NK cells infiltrating a MHC class I-deficient lung adenocarcinoma display impaired cytotoxic activity toward autologous tumor cells associated with altered NK cell-triggering receptors J Immunol 2005 175 5790 5798 16237071 10.4049/jimmunol.175.9.5790

[B39] Kovats S Main EK Librach C Stubblebine M Fisher SJ DeMars R A class I antigen, HLA-G, expressed in human trophoblasts Science 1990 248 220 223 10.1126/science.2326636 2326636

[B40] Le Gal FA Riteau B Sedlik C Khalil-Daher I Menier C Dausset J Guillet JG Carosella ED Rouas-Freiss N HLA-G-mediated inhibition of antigen-specific cytotoxic T lymphocytes Int Immunol 1999 11 1351 1356 10.1093/intimm/11.8.1351 10421792

[B41] Rajagopalan S Long EO A human histocompatibility leukocyte antigen (HLA)-G-specific receptor expressed on all natural killer cells J Exp Med 1999 189 1093 1100 10.1084/jem.189.7.1093 10190900 PMC2193010

[B42] Barrier BF Kendall BS Sharpe-Timms KL Kost ER Characterization of human leukocyte antigen-G (HLA-G) expression in endometrial adenocarcinoma Gynecol Oncol 2006 103 25 30 10.1016/j.ygyno.2006.01.045 16530254

[B43] Ibrahim EC Guerra N Lacombe MJ Angevin E Chouaib S Carosella ED Caignard A Paul P Tumor-specific up-regulation of the nonclassical class I HLA-G antigen expression in renal carcinoma Cancer Res 2001 61 6838 6845 11559559

[B44] Lefebvre S Antoine M Uzan S McMaster M Dausset J Carosella ED Paul P Specific activation of the non-classical class I histocompatibility HLA-G antigen and expression of the ILT2 inhibitory receptor in human breast cancer J Pathol 2002 196 266 274 10.1002/path.1039 11857488

[B45] Ye SR Yang H Li K Dong DD Lin XM Yie SM Human leukocyte antigen G expression: as a significant prognostic indicator for patients with colorectal cancer Mod Pathol 2007 20 375 383 10.1038/modpathol.3800751 17277760

[B46] Belluco C Esposito G Bertorelle R Alaggio R Giacomelli L Bianchi LC Nitti D Lise M Fas ligand is up-regulated during the colorectal adenoma-carcinoma sequence Eur J Surg Oncol 2002 28 120 125 10.1053/ejso.2001.1223 11884046

[B47] Shimoyama M Kanda T Liu L Koyama Y Suda T Sakai Y Hatakeyama K Expression of Fas ligand is an early event in colorectal carcinogenesis J Surg Oncol 2001 76 63 68 10.1002/1096-9098(200101)76:1<63::AID-JSO1011>3.0.CO;2-C 11223827

[B48] Nozoe T Yasuda M Honda M Inutsuka S Korenaga D Fas ligand expression is correlated with metastasis in colorectal carcinoma Oncology 2003 65 83 88 10.1159/000071208 12837986

[B49] Shiraki K Tsuji N Shioda T Isselbacher KJ Takahashi H Expression of Fas ligand in liver metastases of human colonic adenocarcinomas Proc Natl Acad Sci USA 1997 94 6420 6425 10.1073/pnas.94.12.6420 9177233 PMC21065

[B50] Wolkersdorfer GW Marx C Brown J Schroder S Fussel M Rieber EP Kuhlisch E Ehninger G Bornstein SR Prevalence of HLA-DRB1 genotype and altered Fas/Fas ligand expression in adrenocortical carcinoma J Clin Endocrinol Metab 2005 90 1768 1774 10.1210/jc.2004-1406 15585555

[B51] Chopin D Barei-Moniri R Maille P Le Frere-Belda MA Muscatelli-Groux B Merendino N Lecerf L Stoppacciaro A Velotti F Human urinary bladder transitional cell carcinomas acquire the functional Fas ligand during tumor progression Am J Pathol 2003 162 1139 1149 12651606 10.1016/S0002-9440(10)63910-7 PMC1851234

[B52] Korkolopoulou P Goudopoulou A Voutsinas G Thomas-Tsagli E Kapralos P Patsouris E Saetta AA c-FLIP expression in bladder urothelial carcinomas: its role in resistance to Fas-mediated apoptosis and clinicopathologic correlations Urology 2004 63 1198 1204 10.1016/j.urology.2004.01.007 15183989

[B53] Ohta T Elnemr A Kitagawa H Kayahara M Takamura H Fujimura T Nishimura G Shimizu K Yi SQ Miwa K Fas ligand expression in human pancreatic cancer Oncol Rep 2004 12 749 754 15375495

[B54] Ho SY Guo HR Chen HH Hsiao JR Jin YT Tsai ST Prognostic implications of Fas-ligand expression in nasopharyngeal carcinoma Head Neck 2004 26 977 983 10.1002/hed.20090 15390195

[B55] Osaki M Kase S Kodani I Watanabe M Adachi H Ito H Expression of Fas and Fas ligand in human gastric adenomas and intestinal-type carcinomas: correlation with proliferation and apoptosis Gastric Cancer 2001 4 198 205 10.1007/s10120-001-8010-z 11846063

[B56] Kase H Aoki Y Tanaka K Fas ligand expression in cervical adenocarcinoma: relevance to lymph node metastasis and tumor progression Gynecol Oncol 2003 90 70 74 10.1016/S0090-8258(03)00206-3 12821344

[B57] Younes M Schwartz MR Ertan A Finnie D Younes A Fas ligand expression in esophageal carcinomas and their lymph node metastases Cancer 2000 88 524 528 10.1002/(SICI)1097-0142(20000201)88:3<524::AID-CNCR5>3.0.CO;2-U 10649242

[B58] Bennett MW O'Connell J O'Sullivan GC Roche D Brady C Kelly J Collins JK Shanahan F Expression of Fas ligand by human gastric adenocarcinomas: a potential mechanism of immune escape in stomach cancer Gut 1999 44 156 162 10.1136/gut.44.2.156 9895372 PMC1727385

[B59] Bernstorff WV Glickman JN Odze RD Farraye FA Joo HG Goedegebuure PS Eberlein TJ Fas (CD95/APO-1) and Fas ligand expression in normal pancreas and pancreatic tumors. Implications for immune privilege and immune escape Cancer 2002 94 2552 2560 10.1002/cncr.10549 12173320

[B60] Ibrahim R Frederickson H Parr A Ward Y Moncur J Khleif SN Expression of FasL in squamous cell carcinomas of the cervix and cervical intraepithelial neoplasia and its role in tumor escape mechanism Cancer 2006 106 1065 1077 10.1002/cncr.21697 16456813

[B61] O'Connell J Bennett MW O'Sullivan GC Roche D Kelly J Collins JK Shanahan F Fas ligand expression in primary colon adenocarcinomas: evidence that the Fas counterattack is a prevalent mechanism of immune evasion in human colon cancer J Pathol 1998 186 240 246 10211111 10.1002/(SICI)1096-9896(199811)186:3<240::AID-PATH173>3.0.CO;2-L

[B62] Gastman BR Atarshi Y Reichert TE Saito T Balkir L Rabinowich H Whiteside TL Fas ligand is expressed on human squamous cell carcinomas of the head and neck, and it promotes apoptosis of T lymphocytes Cancer Res 1999 59 5356 5364 10537320

[B63] Niehans GA Brunner T Frizelle SP Liston JC Salerno CT Knapp DJ Green DR Kratzke RA Human lung carcinomas express Fas ligand Cancer Res 1997 57 1007 1012 9067260

[B64] Perabo FG Kamp S Schmidt D Lindner H Steiner G Mattes RH Wirger A Pegelow K Albers P Kohn EC von Ruecker A Mueller SC Bladder cancer cells acquire competent mechanisms to escape Fas-mediated apoptosis and immune surveillance in the course of malignant transformation Br J Cancer 2001 84 1330 1338 10.1054/bjoc.2001.1808 11355943 PMC2363638

[B65] Strand S Hofmann WJ Hug H Muller M Otto G Strand D Mariani SM Stremmel W Krammer PH Galle PR Lymphocyte apoptosis induced by CD95 (APO-1/Fas) ligand-expressing tumor cells-a mechanism of immune evasion? Nat Med 1996 2 1361 1366 10.1038/nm1296-1361 8946836

[B66] Ungefroren H Voss M Jansen M Roeder C Henne-Bruns D Kremer B Kalthoff H Human pancreatic adenocarcinomas express Fas and Fas ligand yet are resistant to Fas-mediated apoptosis Cancer Res 1998 58 1741 1749 9563493

[B67] Nagashima H Mori M Sadanaga N Mashino K Yoshikawa Y Sugimachi K Expression of Fas ligand in gastric carcinoma relates to lymph node metastasis Int J Oncol 2001 18 1157 1162 11351245 10.3892/ijo.18.6.1157

[B68] Okada K Komuta K Hashimoto S Matsuzaki S Kanematsu T Koji T Frequency of apoptosis of tumor-infiltrating lymphocytes induced by fas counterattack in human colorectal carcinoma and its correlation with prognosis Clin Cancer Res 2000 6 3560 3564 10999744

[B69] Shimonishi T Isse K Shibata F Aburatani I Tsuneyama K Sabit H Harada K Miyazaki K Nakanuma Y Up-regulation of fas ligand at early stages and down-regulation of Fas at progressed stages of intrahepatic cholangiocarcinoma reflect evasion from immune surveillance Hepatology 2000 32 4 Pt 1 761 769 10.1053/jhep.2000.18192 11003620

[B70] Bennett MW O'Connell J O'Sullivan GC Brady C Roche D Collins JK Shanahan F The Fas counterattack in vivo: apoptotic depletion of tumor-infiltrating lymphocytes associated with Fas ligand expression by human esophageal carcinoma J Immunol 1998 160 5669 5675 9605174

[B71] Houston A Waldron-Lynch FD Bennett MW Roche D O'Sullivan GC Shanahan F O'Connell J Fas ligand expressed in colon cancer is not associated with increased apoptosis of tumor cells in vivo Int J Cancer 2003 107 209 214 10.1002/ijc.11392 12949796

[B72] Ryan AE Shanahan F O'Connell J Houston AM Addressing the "Fas counterattack" controversy: blocking fas ligand expression suppresses tumor immune evasion of colon cancer in vivo Cancer Res 2005 65 9817 9823 10.1158/0008-5472.CAN-05-1462 16267003

[B73] Nishimatsu H Takeuchi T Ueki T Kajiwara T Moriyama N Ishida T Li B Kakizoe T Kitamura T CD95 ligand expression enhances growth of murine renal cell carcinoma in vivo Cancer Immunol Immunother 1999 48 56 61 10.1007/s002620050548 10235489 PMC11037189

[B74] Wada A Tada Y Kawamura K Takiguchi Y Tatsumi K Kuriyama T Takenouchi T O-Wang J Tagawa M The effects of FasL on inflammation and tumor survival are dependent on its expression levels Cancer Gene Ther 2007 14 262 267 10.1038/sj.cgt.7701008 17053813

[B75] Cefai D Schwaninger R Balli M Brunner T Gimmi CD Functional characterization of Fas ligand on tumor cells escaping active specific immunotherapy Cell Death Differ 2001 8 687 695 10.1038/sj.cdd.4400862 11464213

[B76] Dutsch-Wicherek M Tomaszewska R Lazar A Wicherek L Skladzien J The association between RCAS1 expression in laryngeal and pharyngeal cancer and its healthy stroma with cancer relapse BMC Cancer 2009 9 35 10.1186/1471-2407-9-35 19175908 PMC2639609

[B77] Fukuda M Tanaka A Hamao A Suzuki S Kusama K Sakashita H Expression of RCAS1 and its function in human squamous cell carcinoma of the oral cavity Oncol Rep 2004 12 259 267 15254686

[B78] Giaginis C Davides D Zarros A Noussia O Zizi-Serbetzoglou A Kouraklis G Theocharis S Clinical significance of tumor-associated antigen RCAS1 expression in human pancreatic ductal adenocarcinoma Dig Dis Sci 2008 53 1728 1734 10.1007/s10620-007-0035-7 17932753

[B79] Kato H Nakajima M Masuda N Faried A Sohda M Fukai Y Miyazaki T Fukuchi M Tsukada K Kuwano H Expression of RCAS1 in esophageal squamous cell carcinoma is associated with a poor prognosis J Surg Oncol 2005 90 89 94 10.1002/jso.20249 15844180

[B80] Toyoshima T Nakamura S Kumamaru W Kawamura E Ishibashi H Hayashida JN Moriyama M Ohyama Y Sasaki M Shirasuna K Expression of tumor-associated antigen RCAS1 and its possible involvement in immune evasion in oral squamous cell carcinoma J Oral Pathol Med 2006 35 361 368 10.1111/j.1600-0714.2006.00442.x 16762017

[B81] Tsujitani S Saito H Oka S Sakamoto T Kanaji S Tatebe S Ikeguchi M Prognostic significance of RCAS1 expression in relation to the infiltration of dendritic cells and lymphocytes in patients with esophageal carcinoma Dig Dis Sci 2007 52 549 554 10.1007/s10620-006-9408-6 17211709

[B82] Diegmann J Junker K Loncarevic IF Michel S Schimmel B von Eggeling F Immune escape for renal cell carcinoma: CD70 mediates apoptosis in lymphocytes Neoplasia 2006 8 933 938 10.1593/neo.06451 17132225 PMC1716012

[B83] Friedman E Gold LI Klimstra D Zeng ZS Winawer S Cohen A High levels of transforming growth factor beta 1 correlate with disease progression in human colon cancer Cancer Epidemiol Biomarkers Prev 1995 4 549 554 7549813

[B84] Mitropoulos D Kiroudi A Christelli E Serafetinidis E Zervas A Anastasiou I Dimopoulos C Expression of transforming growth factor beta in renal cell carcinoma and matched non-involved renal tissue Urol Res 2004 32 317 322 10.1007/s00240-003-0360-z 15365652

[B85] Santin AD Hermonat PL Hiserodt JC Fruehauf J Schranz V Barclay D Pecorelli S Parham GP Differential transforming growth factor-beta secretion in adenocarcinoma and squamous cell carcinoma of the uterine cervix Gynecol Oncol 1997 64 477 480 10.1006/gyno.1996.4579 9062154

[B86] Walker RA Dearing SJ Transforming growth factor beta 1 in ductal carcinoma in situ and invasive carcinomas of the breast Eur J Cancer 1992 28 641 644 10.1016/S0959-8049(05)80116-9 1317202

[B87] Steiner MS Zhou ZZ Tonb DC Barrack ER Expression of transforming growth factor-beta 1 in prostate cancer Endocrinology 1994 135 2240 2247 10.1210/en.135.5.2240 7956947

[B88] Hazelbag S Gorter A Kenter GG van den Broek L Fleuren G Transforming growth factor-beta1 induces tumor stroma and reduces tumor infiltrate in cervical cancer Hum Pathol 2002 33 1193 1199 10.1053/hupa.2002.130109 12514788

[B89] Halliday GM Le S Transforming growth factor-beta produced by progressor tumors inhibits, while IL-10 produced by regressor tumors enhances, Langerhans cell migration from skin Int Immunol 2001 13 1147 1154 10.1093/intimm/13.9.1147 11526095

[B90] Weber F Byrne SN Le S Brown DA Breit SN Scolyer RA Halliday GM Transforming growth factor-beta1 immobilises dendritic cells within skin tumours and facilitates tumour escape from the immune system Cancer Immunol Immunother 2005 54 898 906 10.1007/s00262-004-0652-3 15776284 PMC11033026

[B91] Huang F Newman E Theodorescu D Kerbel RS Friedman E Transforming growth factor beta 1 (TGF beta 1) is an autocrine positive regulator of colon carcinoma U9 cells in vivo as shown by transfection of a TGF beta 1 antisense expression plasmid Cell Growth Differ 1995 6 1635 1642 9019169

[B92] Demydenko D Berest I Expression of galectin-1 in malignant tumors Exp Oncol 2009 31 74 79 19550395

[B93] Cooper D Ilarregui JM Pesoa SA Croci DO Perretti M Rabinovich GA Multiple functional targets of the immunoregulatory activity of galectin-1: Control of immune cell trafficking, dendritic cell physiology, and T-cell fate Methods Enzymol 2010 480 199 244 full_text 20816212 10.1016/S0076-6879(10)80011-4

[B94] Jung EJ Moon HG Cho BI Jeong CY Joo YT Lee YJ Hong SC Choi SK Ha WS Kim JW Lee CW Lee JS Park ST Galectin-1 expression in cancer-associated stromal cells correlates tumor invasiveness and tumor progression in breast cancer Int J Cancer 2007 120 2331 2338 10.1002/ijc.22434 17304502

[B95] Saussez S Decaestecker C Lorfevre F Cucu DR Mortuaire G Chevalier D Wacreniez A Kaltner H André S Toubeau G Camby I Gabius HJ Kiss R High level of galectin-1 expression is a negative prognostic predictor of recurrence in laryngeal squamous cell carcinomas Int J Oncol 2007 30 1109 1117 17390012

[B96] Spano D Russo R Di Maso V Rosso N Terracciano LM Roncalli M Tornillo L Capasso M Tiribelli C Iolascon A Galectin-1 and its involvement in hepatocellular carcinoma aggressiveness Mol Med 2010 16 102 115 10.2119/molmed.2009.00119 20200618 PMC2829614

[B97] Chiang WF Liu SY Fang LY Lin CN Wu MH Chen YC Chen YL Jin YT Overexpression of galectin-1 at the tumor invasion front is associated with poor prognosis in early-stage oral squamous cell carcinoma Oral Oncol 2008 44 325 334 10.1016/j.oraloncology.2007.03.004 17588803

[B98] Le QT Shi G Cao H Nelson DW Wang Y Chen EY Zhao S Kong C Richardson D O'Byrne KJ Giaccia AJ Koong AC Galectin-1: a link between tumor hypoxia and tumor immune privilege J Clin Oncol 2005 23 8932 8941 10.1200/JCO.2005.02.0206 16219933 PMC12422022

[B99] Kovács-Sólyom F Blaskó A Fajka-Boja R Katona RL Végh L Novák J Szebeni GJ Krenács L Uher F Tubak V Kiss R Monostori E Mechanism of tumor cell-induced T-cell apoptosis mediated by galectin-1 Immunol Lett 2010 127 108 118 19874850 10.1016/j.imlet.2009.10.003

[B100] Dong H Zhu G Tamada K Chen L B7-H1, a third member of the B7 family, co-stimulates T-cell proliferation and interleukin-10 secretion Nat Med 1999 5 1365 1369 10.1038/70932 10581077

[B101] Suh WK Gajewska BU Okada H Gronski MA Bertram EM Dawicki W Duncan GS Bukczynski J Plyte S Elia A Wakeham A Itie A Chung S Da Costa J Arya S Horan T Campbell P Gaida K Ohashi PS Watts TH Yoshinaga SK Bray MR Jordana M Mak TW The B7 family member B7-H3 preferentially down-regulates T helper type 1-mediated immune responses Nat Immunol 2003 4 899 906 10.1038/ni967 12925852

[B102] Sica G Zelano G Settesoldi D Iacopino F Regulation of prostate-specific antigen gene expression by an LH-RH analogue in human prostatic cells Anticancer Res 2003 23 1283 1287 12820384

[B103] Geng L Huang D Liu J Qian Y Deng J Li D Hu Z Zhang J Jiang G Zheng S B7-H1 up-regulated expression in human pancreatic carcinoma tissue associates with tumor progression J Cancer Res Clin Oncol 2008 134 1021 1027 10.1007/s00432-008-0364-8 18347814 PMC12160751

[B104] Nomi T Sho M Akahori T Hamada K Kubo A Kanehiro H Nakamura S Enomoto K Yagita H Azuma M Nakajima Y Clinical significance and therapeutic potential of the programmed death-1 ligand/programmed death-1 pathway in human pancreatic cancer Clin Cancer Res 2007 13 2151 2157 10.1158/1078-0432.CCR-06-2746 17404099

[B105] Krambeck AE Dong H Thompson RH Kuntz SM Lohse CM Leibovich BC Blute ML Sebo TJ Cheville JC Parker AS Kwon ED Survivin and B7-H1 are collaborative predictors of survival and represent potential therapeutic targets for patients with renal cell carcinoma Clin Cancer Res 2007 13 1749 1756 10.1158/1078-0432.CCR-06-2129 17363528

[B106] Thompson RH Kuntz SM Leibovich BC Dong H Lohse CM Webster WS Sengupta S Frank I Parker AS Zincke H Blute ML Sebo TJ Cheville JC Kwon ED Tumor B7-H1 is associated with poor prognosis in renal cell carcinoma patients with long-term follow-up Cancer Res 2006 66 3381 3385 10.1158/0008-5472.CAN-05-4303 16585157

[B107] Gao Q Wang XY Qiu SJ Yamato I Sho M Nakajima Y Zhou J Li BZ Shi YH Xiao YS Xu Y Fan J Overexpression of PD-L1 significantly associates with tumor aggressiveness and postoperative recurrence in human hepatocellular carcinoma Clin Cancer Res 2009 15 971 979 10.1158/1078-0432.CCR-08-1608 19188168

[B108] Wu K Kryczek I Chen L Zou W Welling TH Kupffer cell suppression of CD8^+ ^T cells in human hepatocellular carcinoma is mediated by B7-H1/programmed death-1 interactions Cancer Res 2009 69 8067 8075 10.1158/0008-5472.CAN-09-0901 19826049 PMC4397483

[B109] Boorjian SA Sheinin Y Crispen PL Farmer SA Lohse CM Kuntz SM Leibovich BC Kwon ED Frank I T-cell coregulatory molecule expression in urothelial cell carcinoma: clinicopathologic correlations and association with survival Clin Cancer Res 2008 14 4800 4808 10.1158/1078-0432.CCR-08-0731 18676751

[B110] Konishi J Yamazaki K Azuma M Kinoshita I Dosaka-Akita H Nishimura M B7-H1 expression on non-small cell lung cancer cells and its relationship with tumor-infiltrating lymphocytes and their PD-1 expression Clin Cancer Res 2004 10 5094 5100 10.1158/1078-0432.CCR-04-0428 15297412

[B111] Sun Y Wang Y Zhao J Gu M Giscombe R Lefvert AK Wang X B7-H3 and B7-H4 expression in non-small-cell lung cancer Lung Cancer 2006 53 143 151 10.1016/j.lungcan.2006.05.012 16782226

[B112] Mugler KC Singh M Tringler B Torkko KC Liu W Papkoff J Shroyer KR B7-H4 expression in a range of breast pathology: correlation with tumor T-cell infiltration Appl Immunohistochem Mol Morphol 2007 15 363 370 10.1097/01.pai.0000213159.79557.71 18091377

[B113] Tringler B Zhuo S Pilkington G Torkko KC Singh M Lucia MS Heinz DE Papkoff J Shroyer KR B7-H4 is highly expressed in ductal and lobular breast cancer Clin Cancer Res 2005 11 1842 1848 10.1158/1078-0432.CCR-04-1658 15756008

[B114] Miyatake T Tringler B Liu W Liu SH Papkoff J Enomoto T Torkko KC Dehn DL Swisher A Shroyer KR B7-H4 (DD-O110) is overexpressed in high risk uterine endometrioid adenocarcinomas and inversely correlated with tumor T-cell infiltration Gynecol Oncol 2007 106 119 127 10.1016/j.ygyno.2007.03.039 17509674

[B115] Thompson RH Dong H Kwon ED Implications of B7-H1 expression in clear cell carcinoma of the kidney for prognostication and therapy Clin Cancer Res 2007 13 2 Pt 2 709s 715s 10.1158/1078-0432.CCR-06-1868 17255298

[B116] Shi F Shi M Zeng Z Qi RZ Liu ZW Zhang JY Yang YP Tien P Wang FS PD-1 and PD-L1 upregulation promotes CD8^+ ^T-cell apoptosis and postoperative recurrence in hepatocellular carcinoma patients Int J Cancer 2011 128 887 896 10.1002/ijc.25397 20473887

[B117] Sfanos KS Bruno TC Meeker AK De Marzo AM Isaacs WB Drake CG Human prostate-infiltrating CD8^+ ^T lymphocytes are oligoclonal and PD-1^+^ Prostate 2009 69 1694 1703 10.1002/pros.21020 19670224 PMC2782577

[B118] Matsuzaki J Gnjatic S Mhawech-Fauceglia P Beck A Miller A Tsuji T Eppolito C Qian F Lele S Shrikant P Old LJ Odunsi K Tumor-infiltrating NY-ESO-1-specific CD8^+ ^T cells are negatively regulated by LAG-3 and PD-1 in human ovarian cancer Proc Natl Acad Sci USA 2010 107 7875 7880 10.1073/pnas.1003345107 20385810 PMC2867907

[B119] Zhang Y Huang S Gong D Qin Y Shen Q Programmed death-1 upregulation is correlated with dysfunction of tumor-infiltrating CD8^+ ^T lymphocytes in human non-small cell lung cancer Cell Mol Immunol 2010 7 389 395 10.1038/cmi.2010.28 20514052 PMC4002677

[B120] Munn DH Mellor AL Indoleamine 2,3-dioxygenase and tumor-induced tolerance J Clin Invest 2007 117 1147 1154 10.1172/JCI31178 17476344 PMC1857253

[B121] Ozaki Y Edelstein MP Duch DS Induction of indoleamine 2,3-dioxygenase: a mechanism of the antitumor activity of interferon gamma Proc Natl Acad Sci USA 1988 85 1242 1246 10.1073/pnas.85.4.1242 3124115 PMC279743

[B122] Witkiewicz A Williams TK Cozzitorto J Durkan B Showalter SL Yeo CJ Brody JR Expression of indoleamine 2,3-dioxygenase in metastatic pancreatic ductal adenocarcinoma recruits regulatory T cells to avoid immune detection J Am Coll Surg 2008 206 849 854 10.1016/j.jamcollsurg.2007.12.014 18471709

[B123] Pan K Wang H Chen MS Zhang HK Weng DS Zhou J Huang W Li JJ Song HF Xia JC Expression and prognosis role of indoleamine 2,3-dioxygenase in hepatocellular carcinoma J Cancer Res Clin Oncol 2008 134 1247 1253 10.1007/s00432-008-0395-1 18438685 PMC12161736

[B124] Brandacher G Perathoner A Ladurner R Schneeberger S Obrist P Winkler C Werner ER Werner-Felmayer G Weiss HG Göbel G Margreiter R Königsrainer A Fuchs D Amberger A Prognostic value of indoleamine 2,3-dioxygenase expression in colorectal cancer: effect on tumor-infiltrating T cells Clin Cancer Res 2006 12 1144 1151 10.1158/1078-0432.CCR-05-1966 16489067

[B125] Ino K Yoshida N Kajiyama H Shibata K Yamamoto E Kidokoro K Takahashi N Terauchi M Nawa A Nomura S Nagasaka T Takikawa O Kikkawa F Indoleamine 2,3-dioxygenase is a novel prognostic indicator for endometrial cancer Br J Cancer 2006 95 1555 1561 10.1038/sj.bjc.6603477 17117179 PMC2360726

[B126] Takao M Okamoto A Nikaido T Urashima M Takakura S Saito M Saito M Okamoto S Takikawa O Sasaki H Yasuda M Ochiai K Tanaka T Increased synthesis of indoleamine-2,3-dioxygenase protein is positively associated with impaired survival in patients with serous-type, but not with other types of, ovarian cancer Oncol Rep 2007 17 1333 1339 17487387

[B127] Yoshida N Ino K Ishida Y Kajiyama H Yamamoto E Shibata K Terauchi M Nawa A Akimoto H Takikawa O Isobe K Kikkawa F Overexpression of indoleamine 2,3-dioxygenase in human endometrial carcinoma cells induces rapid tumor growth in a mouse xenograft model Clin Cancer Res 2008 14 7251 7259 10.1158/1078-0432.CCR-08-0991 19010841

[B128] Wu G Morris SM Jr Arginine metabolism: nitric oxide and beyond Biochem J 1998 336 Pt 1 1 17 9806879 10.1042/bj3360001 PMC1219836

[B129] Rodriguez PC Zea AH Culotta KS Zabaleta J Ochoa JB Ochoa AC Regulation of T cell receptor CD3zeta chain expression by L-arginine J Biol Chem 2002 277 21123 21129 10.1074/jbc.M110675200 11950832

[B130] Rodriguez PC Zea AH DeSalvo J Culotta KS Zabaleta J Quiceno DG Ochoa JB Ochoa AC L-arginine consumption by macrophages modulates the expression of CD3 zeta chain in T lymphocytes J Immunol 2003 171 1232 1239 12874210 10.4049/jimmunol.171.3.1232

[B131] Harris BE Pretlow TP Bradley EL Jr Whitehurst GB Pretlow TG Arginase activity in prostatic tissue of patients with benign prostatic hyperplasia and prostatic carcinoma Cancer Res 1983 43 3008 3012 6189588

[B132] Shukla VK Tandon A Ratha BK Sharma D Singh TB Basu S Arginase activity in carcinoma of the gallbladder: a pilot study Eur J Cancer Prev 2009 18 199 202 10.1097/CEJ.0b013e32832405eb 19282757

[B133] Rotondo R Mastracci L Piazza T Barisione G Fabbi M Cassanello M Costa R Morandi B Astigiano S Cesario A Sormani MP Ferlazzo G Grossi F Ratto GB Ferrini S Frumento G Arginase 2 is expressed by human lung cancer, but it neither induces immune suppression, nor affects disease progression Int J Cancer 2008 123 1108 1116 10.1002/ijc.23437 18528866

[B134] Suer Gokmen S Yoruk Y Cakir E Yorulmaz F Gulen S Arginase and ornithine, as markers in human non-small cell lung carcinoma Cancer Biochem Biophys 1999 17 125 131 10738908

[B135] Bronte V Kasic T Gri G Gallana K Borsellino G Marigo I Battistini L Iafrate M Prayer-Galetti T Pagano F Viola A Boosting antitumor responses of T lymphocytes infiltrating human prostate cancers J Exp Med 2005 201 1257 1268 10.1084/jem.20042028 15824085 PMC2213151

[B136] Esendagli G Bruderek K Goldmann T Busche A Branscheid D Vollmer E Brandau S Malignant and non-malignant lung tissue areas are differentially populated by natural killer cells and regulatory T cells in non-small cell lung cancer Lung Cancer 2008 59 32 40 10.1016/j.lungcan.2007.07.022 17825949

[B137] Griffiths RW Elkord E Gilham DE Ramani V Clarke N Stern PL Hawkins RE Frequency of regulatory T cells in renal cell carcinoma patients and investigation of correlation with survival Cancer Immunol Immunother 2007 56 1743 1753 10.1007/s00262-007-0318-z 17487490 PMC11030591

[B138] Hiraoka N Onozato K Kosuge T Hirohashi S Prevalence of FOXP3^+ ^regulatory T cells increases during the progression of pancreatic ductal adenocarcinoma and its premalignant lesions Clin Cancer Res 2006 12 5423 5434 10.1158/1078-0432.CCR-06-0369 17000676

[B139] Kobayashi N Hiraoka N Yamagami W Ojima H Kanai Y Kosuge T Nakajima A Hirohashi S FOXP3^+ ^regulatory T cells affect the development and progression of hepatocarcinogenesis Clin Cancer Res 2007 13 902 911 10.1158/1078-0432.CCR-06-2363 17289884

[B140] Liyanage UK Moore TT Joo HG Tanaka Y Herrmann V Doherty G Drebin JA Strasberg SM Eberlein TJ Goedegebuure PS Linehan DC Prevalence of regulatory T cells is increased in peripheral blood and tumor microenvironment of patients with pancreas or breast adenocarcinoma J Immunol 2002 169 2756 2761 12193750 10.4049/jimmunol.169.5.2756

[B141] Schwarz S Butz M Morsczeck C Reichert TE Driemel O Increased number of CD25 FoxP3 regulatory T cells in oral squamous cell carcinomas detected by chromogenic immunohistochemical double staining J Oral Pathol Med 2008 37 485 489 10.1111/j.1600-0714.2008.00641.x 18355177

[B142] Siddiqui SA Frigola X Bonne-Annee S Mercader M Kuntz SM Krambeck AE Sengupta S Dong H Cheville JC Lohse CM Krco CJ Tumor-infiltrating Foxp3^-^CD4^+^CD25^+ ^T cells predict poor survival in renal cell carcinoma Clin Cancer Res 2007 13 2075 2081 10.1158/1078-0432.CCR-06-2139 17404089

[B143] Viehl CT Moore TT Liyanage UK Frey DM Ehlers JP Eberlein TJ Goedegebuure PS Linehan DC Depletion of CD4^+^CD25^+ ^regulatory T cells promotes a tumor-specific immune response in pancreas cancer-bearing mice Ann Surg Oncol 2006 13 1252 1258 10.1245/s10434-006-9015-y 16952047

[B144] Kaporis HG Guttman-Yassky E Lowes MA Haider AS Fuentes-Duculan J Darabi K Whynot-Ertelt J Khatcherian A Cardinale I Novitskaya I Krueger JG Carucci JA Human basal cell carcinoma is associated with Foxp3^+ ^T cells in a Th2 dominant microenvironment J Invest Dermatol 2007 127 2391 2398 10.1038/sj.jid.5700884 17508019

[B145] Sharma S Yang SC Zhu L Reckamp K Gardner B Baratelli F Huang M Batra RK Dubinett SM Tumor cyclooxygenase-2/prostaglandin E2-dependent promotion of FOXP3 expression and CD4^+^CD25^+ ^T regulatory cell activities in lung cancer Cancer Res 2005 65 5211 5220 10.1158/0008-5472.CAN-05-0141 15958566

[B146] Curiel TJ Coukos G Zou L Alvarez X Cheng P Mottram P Evdemon-Hogan M Conejo-Garcia JR Zhang L Burow M Zhu Y Wei S Kryczek I Daniel B Gordon A Myers L Lackner A Disis ML Knutson KL Chen L Zou W Specific recruitment of regulatory T cells in ovarian carcinoma fosters immune privilege and predicts reduced survival Nat Med 2004 10 942 949 10.1038/nm1093 15322536

[B147] Tan MC Goedegebuure PS Belt BA Flaherty B Sankpal N Gillanders WE Eberlein TJ Hsieh CS Linehan DC Disruption of CCR5-dependent homing of regulatory T cells inhibits tumor growth in a murine model of pancreatic cancer J Immunol 2009 182 1746 1755 19155524 10.4049/jimmunol.182.3.1746 PMC3738070

[B148] Almand B Resser JR Lindman B Nadaf S Clark JI Kwon ED Carbone DP Gabrilovich DI Clinical significance of defective dendritic cell differentiation in cancer Clin Cancer Res 2000 6 1755 1766 10815894

[B149] Garrity T Pandit R Wright MA Benefield J Keni S Young MR Increased presence of CD34^+ ^cells in the peripheral blood of head and neck cancer patients and their differentiation into dendritic cells Int J Cancer 1997 73 663 669 10.1002/(SICI)1097-0215(19971127)73:5<663::AID-IJC9>3.0.CO;2-V 9398043

[B150] Schmielau J Finn OJ Activated granulocytes and granulocyte-derived hydrogen peroxide are the underlying mechanism of suppression of t-cell function in advanced cancer patients Cancer Res 2001 61 4756 4760 11406548

[B151] Bluth MJ Zaba LC Moussai D Suarez-Farinas M Kaporis H Fan L Pierson KC White TR Pitts-Kiefer A Fuentes-Duculan J Guttman-Yassky E Krueger JG Lowes MA Carucci JA Myeloid dendritic cells from human cutaneous squamous cell carcinoma are poor stimulators of T-cell proliferation J Invest Dermatol 2009 129 2451 2462 10.1038/jid.2009.96 19387481 PMC2846605

[B152] Pak AS Wright MA Matthews JP Collins SL Petruzzelli GJ Young MR Mechanisms of immune suppression in patients with head and neck cancer: presence of CD34^+ ^cells which suppress immune functions within cancers that secrete granulocyte-macrophage colony-stimulating factor Clin Cancer Res 1995 1 95 103 9815891

[B153] Young MR Wright MA Lozano Y Matthews JP Benefield J Prechel MM Mechanisms of immune suppression in patients with head and neck cancer: influence on the immune infiltrate of the cancer Int J Cancer 1996 67 333 338 10.1002/(SICI)1097-0215(19960729)67:3<333::AID-IJC5>3.0.CO;2-S 8707405

[B154] Young MR Wright MA Lozano Y Prechel MM Benefield J Leonetti JP Collins SL Petruzzelli GJ Increased recurrence and metastasis in patients whose primary head and neck squamous cell carcinomas secreted granulocyte-macrophage colony-stimulating factor and contained CD34^+ ^natural suppressor cells Int J Cancer 1997 74 69 74 10.1002/(SICI)1097-0215(19970220)74:1<69::AID-IJC12>3.0.CO;2-D 9036872

[B155] Norian LA Rodriguez PC O'Mara LA Zabaleta J Ochoa AC Cella M Allen PM Tumor-infiltrating regulatory dendritic cells inhibit CD8^+ ^T cell function via L-arginine metabolism Cancer Res 2009 69 3086 3094 10.1158/0008-5472.CAN-08-2826 19293186 PMC2848068

[B156] Hoechst B Voigtlaender T Ormandy L Gamrekelashvili J Zhao F Wedemeyer H Lehner F Manns MP Greten TF Korangy F Myeloid derived suppressor cells inhibit natural killer cells in patients with hepatocellular carcinoma via the NKp30 receptor Hepatology 2009 50 799 807 10.1002/hep.23054 19551844 PMC6357774

[B157] Kusmartsev S Su Z Heiser A Dannull J Eruslanov E Kubler H Yancey D Dahm P Vieweg J Reversal of myeloid cell-mediated immunosuppression in patients with metastatic renal cell carcinoma Clin Cancer Res 2008 14 8270 8278 10.1158/1078-0432.CCR-08-0165 19088044

[B158] Zea AH Rodriguez PC Atkins MB Hernandez C Signoretti S Zabaleta J McDermott D Quiceno D Youmans A O'Neill A Mier J Ochoa AC Arginase-producing myeloid suppressor cells in renal cell carcinoma patients: a mechanism of tumor evasion Cancer Res 2005 65 3044 3048 15833831 10.1158/0008-5472.CAN-04-4505

[B159] Hoechst B Ormandy LA Ballmaier M Lehner F Kruger C Manns MP Greten TF Korangy F A new population of myeloid-derived suppressor cells in hepatocellular carcinoma patients induces CD4^+^CD25^+^Foxp3^+ ^T cells Gastroenterology 2008 135 234 243 10.1053/j.gastro.2008.03.020 18485901

